# IL-6-induced response of human osteoblasts from patients with rheumatoid arthritis after inhibition of the signaling pathway

**DOI:** 10.1007/s10238-023-01103-3

**Published:** 2023-06-07

**Authors:** Marie-Luise Sellin, Annett Klinder, Philipp Bergschmidt, Rainer Bader, Anika Jonitz-Heincke

**Affiliations:** 1 Department of Orthopaedics, Research Laboratory for Biomechanics and Implant Technology, Rostock University Medical Center, Doberaner Strasse 142, 18057 Rostock, Germany; 2 Department for Orthopaedic Surgery, Trauma Surgery and Hand Surgery, Suedstadt Hospital Rostock, Suedring 81, 18059 Rostock, Germany

**Keywords:** Rheumatoid arthritis, Interleukin 6, Sarilumab, Osteoblasts, Bone remodeling, Particles

## Abstract

Interleukin (IL-) 6 is a critical factor in inflammatory processes of rheumatoid arthritis (RA). This is of high interest as the progression of RA may lead to the implantation of joint endoprostheses, which is associated with a pro-inflammatory increase in IL-6 in the periprosthetic tissue. Biological agents such as sarilumab have been developed to inhibit IL-6-mediated signaling. However, IL-6 signaling blockade should consider the inhibition of inflammatory processes and the regenerative functions of IL-6. This in vitro study investigated whether inhibiting IL-6 receptors can affect the differentiation of osteoblasts isolated from patients with RA. Since wear particles can be generated at the articular surfaces of endoprostheses leading to osteolysis and implant loosening, the potential of sarilumab to inhibit wear particle-induced pro-inflammatory processes should be investigated. Both in monocultures and indirect co-cultures with osteoclast-like cells (OLCs), human osteoblasts were stimulated with 50 ng/mL each of IL-6 + sIL-6R and in combination with sarilumab (250 nM) to characterize cell viability and osteogenic differentiation capacity. Furthermore, the influence of IL-6 + sIL-6R or sarilumab on viability, differentiation, and inflammation was evaluated in osteoblasts exposed to particles. Stimulation with IL-6 + sIL-6R and sarilumab did not affect cell viability. Except for the significant induction of *RUNX2* mRNA by IL-6 + sIL-6R and a significant reduction with sarilumab, no effects on cell differentiation and mineralization could be detected. Furthermore, the different stimulations did not affect the osteogenic and osteoclastic differentiation of co-cultured cells. Compared to the osteoblastic monocultures, a decreased release of IL-8 was triggered in the co-culture. Among these, treatment with sarilumab alone resulted in the greatest reduction of IL-8. The co-culture also showed clearly increased OPN concentrations than the respective monocultures, with OPN secretion apparently triggered by the OLCs. Particle exposure demonstrated decreased osteogenic differentiation using different treatment strategies. However, sarilumab administration caused a trend toward a decrease in IL-8 production after stimulation with IL-6 + sIL-6R. The blockade of IL-6 and its pathway have no significant effect on the osteogenic and osteoclastic differentiation of bone cells derived from patients with RA. Nonetheless, observed effects on the reduced IL-8 secretion need further investigation.

## Introduction

Rheumatoid arthritis (RA) is a chronic autoimmune disease characterized by symmetric inflammation in the joints that can lead to cartilage destruction and bone erosion. The pathomechanism involves a complex interplay of different cell types, including dendritic cells, T cells, macrophages, B cells, fibroblasts, and osteoclasts. Secretion of RA-specific autoantigens, for example, immunoglobulin (Ig) M and IgG rheumatoid factors or citrullinated peptides [1, 2], leads to persistent activation of immune cells and results in chronic inflammation. The inflammatory response is mediated by various cytokines, most importantly interleukin (IL)-6, which is considered a pro-inflammatory key cytokine [1, 3]. The cytokine synthesis by the cells of the innate immune system is stimulated by the activation of Toll-like receptors (TLR) via IL-1, tumor necrosis factor (TNF)-α, and IL-17. In addition, it has been reported that osteoblasts (hOB), chondrocytes, and synovial fibroblasts are able to secrete IL-6. In joints, IL-6 is involved in bone resorption by osteoclasts, as IL-6 increases the expression of receptor activator of nuclear factor κB (RANK) ligand (RANKL) on osteoblasts and synovial cells, resulting in osteoclast differentiation, and pannus formation [1]. IL-6 exerts its function through the IL-6 receptor (IL-6R), which is composed of a type I cytokine alpha receptor subunit and the signal transducing beta receptor subunit (glycoprotein (gp)130). The receptor exists in two forms: membrane-bound (IL-6R) and soluble (sIL-6R). The membrane-bound receptor is expressed mainly by T cells, B cells, monocytes, osteocytes, and osteoblasts, whereas the soluble form is formed via differential splicing or by cleavage of the extracellular domain of IL-6R [4]. The signaling cascade of IL-6 can be activated by various receptor-ligand interactions. In classical signaling (cis-signaling), IL-6 binds to the alpha subunit of the IL-6R, which is mainly expressed on cells of lymphoid or myeloid lineage [5] and initiates the acute phase response via gp130 but is also involved in tissue regeneration [6]. IL-6 can also bind to the alpha subunit of sIL-6R, allowing the resulting complex to bind to gp130 of endothelial cells or synovial fibroblasts not expressing IL-6R, thereby inducing the pro-inflammatory effect with monocyte recruitment and macrophage differentiation, as well as T-cell recruitment and differentiation [6–8]. The receptor-ligand interaction leads to Janus kinase (JAK) activation. JAK activates signal transducer and activator of transcription (STAT) family 3, which translocates to the nucleus where it acts as a transcription factor [5]. Because this signaling pathway has a major impact on cellular differentiation, growth, and osteoclastogenesis, several drugs have been developed to modulate the pathway [2, 9]. Therapeutic approaches for RA include disease-modifying anti-rheumatic drugs (DMARDs) designed to reduce inflammation and thus prevent further joint damage. They can be divided into three groups: (i) conventional synthetic DMARDs (methotrexate [MTX]), (ii) targeted synthetic DMARDs (JAK1/2-inhibitors), and (iii) biologic DMARDs (TNF-α inhibitors, TNF-receptor inhibitors) [2]. Sarilumab is a monoclonal antibody that can bind to both forms of IL-6R. Consequently, signal transduction of IL-6 in cells is inhibited, the inflammatory response is reduced, and bone and cartilage are protected from destruction [7]. The antibody was approved in combination with MTX but showed also a good efficacy when used as monotherapy [1], making it an alternative therapeutic option in MTX-intolerant patients and non-responders [10].

In a previous study, we looked at the impact of the IL-6 blockade by sarilumab on osteogenic differentiation. We could show that co-stimulation of IL-6 and sIL-6R enhanced osteogenic differentiation in osteoblasts derived from osteoarthritic patients. This effect was attenuated by the simultaneous treatment with sarilumab [11].

The present study was designed to investigate further the influence of IL-6 in combination with sIL-6R and sarilumab on osteoblasts derived from patients with rheumatoid arthritis. This is particularly interesting when joint arthroplasty becomes necessary due to RA-related joint destruction. The implantation of endoprostheses is associated with an increase in IL-6 concentration, thus inducing inflammation within the periprosthetic tissue [12]. However, implant ingrowth into surrounding bone tissue occurs with the activation of an osteogenic signaling cascade of intracellular and extracellular biological factors, which include an inflammatory phase, a proliferative phase, and a remodeling phase [13, 14]. In particular, the inflammatory phase immediately after implant insertion is of particular importance, as released growth factors and cytokines lead to the formation of a blood clot on which lipids and proteins can bind, thus promoting migration and proliferation of cells from the periphery [15]. The inflammatory reaction within the implant site, which may be accompanied by an increase in IL-6, for example, may thus also be regarded as induction for bone formation processes. Therefore, particular attention was paid to the osteogenic differentiation capacity and mineralization as well as the morphology of human osteoblasts derived from RA patients. In addition, indirect co-culture experiments with osteoblasts and osteoclastic cells were performed to investigate the effects of different stimulants on bone remodeling processes. In the long term, however, movements on the articulating implant surfaces or micromotions at the interface between the implant and bone stock can lead to abrasive wear and corrosion, which in turn trigger inflammatory processes, subsequent osteolysis, and implant loosening [16]. Therefore, the potential of sarilumab to inhibit particle-induced pro-inflammatory processes during RA therapy should be analyzed.

## Materials and methods

### Cell culture of human primary osteoblasts

Isolation of human primary osteoblasts was performed according to a well-established protocol by Lochner et al. [17] from femoral heads of patients with RA-related osteoarthritis. The femoral heads were provided after obtaining informed consent from patients undergoing total hip arthroplasty. The study was approved by the local ethical committee, ref. number A2018-0234. A total of seven patients were recruited. The gender and age distribution and the existing medications are summarized in Table 1. The Isolated osteoblasts of patients with RA were used for the stimulation experiments.

**Table 1:** Overview of clinical data of RA patients and the experimental setup

Donor no	Gender, Age	Medication	Experimental setup		
			Cell phenotype	Co-culture	Particle exposure
1	Male, 65	Prednisolone: 2.5 mg 1 × 1, Humira: 20 mg, bi-weekly s.c	x	x	x
2	Female, 68	Methotrexate: 15 mg/0.3 mL (1 × weekly), Prednisolone: 60 mg 1 × 1	x		x
3	Male,66	Prednisolone: 2 mg 1 × 1, Celebrex: 200 mg 1 × 1, MabThera-Infusion: ¾-yearly	x		x
4	Male, 74	Prednisolone: 2.5 mg 1 × 1, MTX Syringe (1 × weekly)	x		x
5	Female, 69	Prednisolone: 2.5 mg 1 × 1, Olumiant: 2 mg 1 × 1	x	x	x
6	Female, 70	Tocilizumab: 162 mg Pen bi-weekly, Prednisolone: 2.5 mg 1 × 1,	x	x	x
7	Female, 78	Prednisolone: 2.5 mg 1 × 1	x		x

The isolated cells were cultured under standard cell culture conditions at 37 °C and 5% CO_2_ at 95% humidity in calcium-free Dulbecco's Modified Eagle Medium (DMEM; PAN-Biotech, Aidenbach, Germany) containing 10% fetal calf serum (FCS; PAN-Biotech, Aidenbach, Germany), 1% amphotericin B, 1% penicillin–streptomycin, and 1% 2-(4-(2-hydroxyethyl)-1-piperazinyl)-ethanesulfonic acid (HEPES buffer; all: Sigma-Aldrich, Munich, Germany). The absence of calcium in the cell culture medium suppresses the mineralization of the osteoblastic cells. To maintain the osteogenic phenotype, 10 mM β-glycerophosphate, 50 μg/ml ascorbic acid, and 100 nM dexamethasone were added to the cell culture medium (all: Sigma-Aldrich, Munich, Germany).

For single culture experiments, cells from passage 3 were seeded in 48-well cell culture plates with 15.000 cells per well and cultured for 24 h to allow cell adherence. Afterward, cells were stimulated with IL-6 (50 ng/ml, Peprotech, Hamburg, Germany) and recombinant human sIL6R (50 ng/ml, CD126; ImmunoTools, Friesoythe; Germany). Based on the dosing regimen (200 mg every two weeks for human application) that was associated with a significant improvement of the symptoms in RA patients, the sarilumab concentration of 250 nM (40 µg/ml, Kevzara®, Sanofi-Aventis, Montpellier, France) was considered for cell culture experiments. All concentrations were based on our previous study [11]. If not otherwise stated, the following stimulation groups were defined: (i) sarilumab, (ii) IL-6 + sIL-6R, (iii) IL-6 + sIL-6R + sarilumab, and iv) unstimulated control.

### Isolation and cultivation of human PBMCs

Human peripheral blood mononuclear cells (PBMCs) were isolated from whole blood of the RA patients (three donors). Isolation was performed using Histopaque®-1077 (Sigma-Aldrich, Munich, Germany) as density gradient and SepMate™-50 (Stemcell™ Technologies, Vancouver, BC, Canada) tubes according to the manufacturer's protocol. The tubes were centrifuged with 1200 × g for 20 min with full acceleration and break on. After isolation, cells, now referred to as mononuclear osteoclast progenitor cells (OPCs), were cultured in cell-repellent 6-well cell culture plates in Roswell Park Memorial Institute (RPMI; PAN-Biotech, Aidenbach, Germany) 1640 supplemented with 1% amphotericin B, 1% penicillin–streptomycin, 20% FCS and 2% stable glutamine (PAN-Biotech, Aidenbach, Germany). After five days, cell supernatant with non-adherent cells was transferred to 50 ml tubes, each well of the plate was rinsed with PBS, and the tubes were centrifuged at 120 × g for 8 min.

For co-culture experiments, cells were seeded in 12-well cell culture plates with 300.000 cells per well and incubated for 14 days. For differentiation of OPCs in polynuclear osteoclast-like cells (OLCs), the medium was supplemented with 5 µg/mL human sRANK-Ligand and 2,5 µg/mL human macrophage colony-stimulating factor (M-CSF; both: Peprotech Inc., Rocky Hill, NJ, USA). After seven days of incubation, the whole medium was replaced.

### Indirect co-culture experiments with osteoblasts and OLCs

Indirect co-culture experiments were performed using semipermeable cell culture inserts with 8 µm pores for 12-well plates (Greiner Bio-One, Frickenhausen, Germany). Osteoblasts were seeded in cell culture inserts with 50,000 cells per insert in DMEM medium supplemented with additives, as mentioned in 2.1. After 24 h of incubation, the inserts were transferred to cell culture plates containing 300.000 OLCs per well. The cells were stimulated with IL-6 (50 ng/mL), recombinant human sIL6R (50 ng/mL), and sarilumab (250 nM) for seven days. Since it was shown that the supplementation of the different cell culture additives, such as M-CSF or dexamethasone, affects the differentiation of the cell types, only ascorbic acid and glutamine were added to the media for these stimulation experiments [18]. RPMI medium used for stimulation was supplemented with 2% FCS and 2% stable glutamine, while 10% FCS and 50 μg/mL ascorbic acid was added to DMEM medium. Indirect co-culture experiments were performed for seven days. Single cultures of human osteoblasts or OLCs under the same conditions served as controls. For the following analyses, the two cell types were examined separately, whereby the secretion of proteins was determined in the total supernatant of both cell types.

### Exposure of osteoblasts to metallic particles

Osteoblasts from RA patients were treated with particles derived from cobalt-chromium-molybdenum (CoCr28Mo6, Continuum Blue, Cardiff, UK) alloyed samples to observe the effect of particle exposure. Particle characteristics were previously described by our group [19, 20]. For this experiment, 15,000 osteoblasts per well were seeded in a 48 well plate. After 24 h, the cells were first stimulated with IL-6 + sIL-6R ± sarilumab and incubated for an additional 24 h. Then, the particle solution was added to the cell culture with a concentration of 0.01 mg/mL. Osteoblasts were exposed to particles for a further 48 h before evaluation. To avoid agglomeration of the metallic particles in stock solution with a concentration of 1 mg/mL, they were stored in ethanol. Therefore, appropriate cell cultures incubated only with ethanol (70% v/v; diluted 1:100 in medium) were included as solvent controls, along with unstimulated cells.

### Determination of cell morphology and viability

The viability of cells was determined by a water-soluble tetrazolium salt (WST-1; Roche, Penzberg, Germany) assay. At the end of the respective incubation time, cells were washed with phosphate-buffered saline (PBS; Biochrom AG, Berlin, Germany). Afterward, they were incubated for 30 min at 37 °C, and 5% CO_2_ with WST-1 reagent was diluted 1:10 with medium. Then, 100 µl supernatant was transferred in duplicates to a 96-well plate, and absorbance was measured at 450 nm (reference 630 nm) in a microplate reader (Tecan Group AG, Maennedorf, Switzerland).

To analyze changes in the structure of the cytoskeleton of the cells after treatment with IL-6 + sIL-6R ± sarilumab, actin staining with diamidino-2-phenylindole dihydrochloride (DAPI) counterstain was performed. For this purpose, the medium was removed, and the cells were washed with PBS. The following steps were performed protected from light and at room temperature. Before staining, cells were fixed with 4% paraformaldehyde (PFA; pH: 7.0). After 10 min, cells were rinsed with PBS for 30 s. Then, the cell membrane was permeabilized by adding a permeabilization buffer containing 0.05% Triton-X (Merck KGaA, Darmstadt, Germany) for 5 min. Osteoblasts were rewashed with PBS for 30 s before 100 nM actin staining solution (100 nM Acti-Stain 488 Fluorescent Phalloidin, Cytoskeleton, Denver, CO, USA) was added to the cells for an incubation time of 30 min. After washing three times with PBS, osteoblasts were incubated with DAPI (Merck KGaA, Darmstadt, Germany) for 5 min to counterstain the nuclei of the cells. The staining solution was removed, and cells were washed with PBS. Stained osteoblasts were embedded and stored for 24 h at 4 °C. Microscopic examinations were performed using the CytoViva® Enhanced Darkfield Hyperspectral Microscope System (CytoViva, Inc., Auburn, AL, USA) and a 60 × oil objective. CytoViva's dual mode fluorescence module allows simultaneous real-time observation of fluorescent and non-fluorescent sample components. The green fluorescent cytoskeleton of the cells was imaged at a wavelength of 525 nm using a bandpass emission filter (69002 m, Chroma Technology Corporation, VT, USA). The blue fluorescence of the nuclei stained with DAPI was recorded at a wavelength of 461 nm. The fluorescence images were taken from the same image section and layered upon each other using image processing software. Image acquisition was performed using *Ocular Imaging* software (Teledyne Photometrics, Tucson, AZ, USA). In addition, images were acquired in the darkfield so that unstained structures such as intracellular vesicles, large endosomes, and granules could be visualized. Additionally, microscopic examinations of cell cultures were carried out after 72 h, seven days, and 14 days of incubation. Further, the morphology of osteoblasts was documented via light microscopy using 400 × magnification (Nikon ECLIPSE TS100, Nikon GmbH, Duesseldorf, Germany).

### Hyperspectral imaging

Hyperspectral images and data were captured using an optical microscope (Olympus BX 41) equipped with an advanced darkfield illumination system and integrated hyperspectral imaging (HSI) spectrometer (CytoViva Inc., Auburn, AL, USA). Spatial and spectral data (wavelengths between 400 and 1000 nm) were collected at 600 × magnification. Detection of particles occurred when the signal for the material in the given pixel was greater than the background noise. Spectral libraries were created by analyzing solutions containing CoCr28Mo6 particles in ethanol. To create a spectral library, pixels that could be identified as particles were selected and marked using the region of interest (ROI) tool. The ROI was converted into a spectral library. After hyperspectral imaging of particles in solution, cells treated with 50 ng/mL IL-6 + 50 ng/mL IL-6R ± 250 nM sarilumab and CoCr28Mo6 particles were imaged. Hyperspectral analysis was performed using Environment for Visualization (ENVI) v4.8 software (Exelis Visual Information Solutions, Boulder, CO, USA).

### Quantification of secreted proteins in cell culture supernatants

Protein levels of markers for osteoblastic differentiation (c-terminal pro-peptide of collagen type 1 (C1CP): MicroVue™ CICP EIA, Quidel, Marburg, Germany; osteopontin (OPN): Human Osteopontin ELISA Kit, Abcam, Cambridge, UK), IL-6 signaling (human sgp 130 ELISA, Sigma-Aldrich, ST. Louis MO, USA; IL-6: Human IL-6 ELISA Ready-SET-Go!®, ThermoFisher Scientific, Waltham, MA, USA; sIL-6R: IL-6 Receptor Human ELISA Kit, Abcam, Cambridge, UK) as well as IL-8 (Human IL-8 ELISA Ready-SET-Go!®, ThermoFisher Scientific, Waltham, MA, USA) were determined in the supernatant of control and exposed osteoblasts via enzyme-linked immunosorbent assay. For this purpose, supernatants were collected and stored at -20 °C before quantification. ELISAs were performed according to the manufacturer's recommendations. Absorbance was measured at 405 nm (reference wavelength: 630 nm; IL-6: 570 nm) using the Tecan Infinite® 200 Pro microplate reader (Tecan Group AG, Maennedorf, Switzerland). The sample concentrations were calculated using a standard curve. Normalization of protein content to total protein was performed using the Qubit Protein Assay Kit and Qubit 1.0 (both: Thermo Fisher Scientific, Waltham, MA, USA) according to the manufacturer's instructions.

### Mineralization assay osteoimage

Osteoblasts' in vitro mineralization capacity was quantified using OsteoImage™ Mineralization Assay (Lonza Inc., Morristown, NJ, USA). For this purpose, the cells were incubated with the medium described in 2.1, which was further supplemented with calcium chloride dehydrate (CaCl_2_*2xH_2_0, final concentration: 1.8 mmol/L). 24 h after seeding, cells were stimulated with IL-6 + IL-6R ± sarilumab in calcium-supplemented medium and incubated for seven or 14 days. Untreated cells incubated with calcium-containing medium served as controls. After seven days, the medium was changed. After the incubation period, the hydroxyapatite of bone-like nodules was stained according to the manufacturer's protocol. First, cells were fixed with 4% PFA and rinsed with a wash buffer. The staining solution was added, and the osteoblasts were incubated protected from light at room temperature. After an incubation period of 30 min, the staining solution was removed, and the wells were washed three times for 5 min with washing buffer. The fluorescence signal of mineralization was measured at 492/520 nm excitation/emission using the microplate reader (Tecan Group AG, Maennedorf, Switzerland). In addition, microscopic images of the mineralization nodules were taken with a fluorescence microscope using a 40 × objective (Nikon ECLIPSE TS100, Nikon GmbH, Duesseldorf, Germany).

### Quantification of enzymatic ALP activity

The activity of enzymatic alkaline phosphatase (ALP) was determined by washing the cells with tris-buffered saline (TBS; 137 nm NaCl, 2.7 M KCl, 50 mM Tris) and lysing them for 10 min at room temperature with aqua dest. containing 1% Tween (Sigma-Aldrich, Munich, Germany) and 1 mM phenylmethylsulfonyl fluoride (PMSF; AppliChem, Darmstadt, Germany). After lysis, cells were treated with 1 mM p-nitrophenyl phosphate, 100 mM 2-amino2-methyl-1-propanol, and 5 mM magnesium chloride (MgCl_2_; both: Sigma-Aldrich, Munich, Germany) in distilled water at 37 °C. After one hour, the reaction was stopped with 2 M NaOH. The absorbance of the solution was measured at 405 nm in a microplate reader (Tecan Group AG, Maennedorf, Switzerland).

### Gene expression analysis

Ribonucleic acid (RNA) was isolated using the innuPREP RNA Mini Kit 2.0 (Analytic Jena GmbH, Jena, Germany) following the manufacturer's protocol. After elution of the RNA into a fresh sterile tube using RNase-free water, RNA concentration was measured using the Tecan Infinite® 200 (Tecan Group AG, Maennedorf, Switzerland) microplate reader and the NanoQuant Plate™. RNase-free water served as blank. Subsequently, the High Capacity cDNA Reverse Transcription Kit (Applied Biosystems, Forster City, CA, USA) was used to transcribe 100 ng of RNA from each sample into complementary deoxyribonucleic acid (cDNA). The PCR was done with the following protocol: 10 min at 25 °C, 120 min at 37 °C, and 5 min at 85 °C in a thermocycler (Analytik Jena GmbH, Jena, Germany). Afterward, samples were diluted in 20 µl RNase-free water and stored at − 20 °C.

Expression levels of differentiation- and inflammation-associated genes were determined by a semi-quantitative real-time polymerase chain reaction (qPCR; qTower 2.0, Analytik Jena GmbH, Jena, Germany) using innuMIX qPCR MasterMix SyGreen (Analytik Jena AG, Jena, Germany) and the primer pairs listed in Table 2. Each sample was measured in duplicate. QPCR was performed under the following protocol: 2 min at 95 °C, followed by 40 times of rotation of denaturation of 5 s at 95 °C, and annealing/elongation for 25 s at 60–65 °C. A cycle threshold (Ct) of 28 was set as a limit. The relative amount of each mRNA compared with the housekeeping gene β-Actin (osteoblasts) or hypoxanthine guanine phosphoribosyl transferase (*HPRT*; OLCs) was calculated by the equation ΔCt = Ct_target_-Ct_housekeeping gene_. The relative expression of target mRNA of unstimulated cells and treated cells was calculated using the 2(^−ΔΔCt^) method (% unstimulated control).

**Table 2:** cDNA target sequences for semi-quantitative real-time PCR

Gene	Forward primer (5' – 3')	Reverse Primer (5’ – 3’)
Alkaline phosphatase (ALPL)	CATTGTGACCACCACGAGAG	CCATGATCACGTCAATGTCC
Cathepsin K (CTSK)	GGAAGCAATATAACAACAAGGTGGA	GGGGCTCTACCTTCCCATTC
Collagen type 1 (Col1A1)	ACGAAGACATCCCACCAATC	AGATCACGTCATCGCACAAC
Glycoprotein 130 (IL6ST)	ACCTATTTAAGAGCACCTTCCAA	TCCCACTCTAAGACAGCTTCG
Hypoxanthine–guanine phosphoribosyl transferase (HPRT)	CCCTGGCGTCGTGATTAGTG	TCGAGCAAGACGTTCAGTCC
Integrin Binding Sialoprotein (IBSP)	ATTTTGGGAATGGCCTGTGC	GTCACTACTGCCCTGAACTGG
Interleukin 8 (IL8)	TCTGTGTGAAGGTGCAGTTTTG	ATTTCTGTGTTGGCGCAGTG
Matrix-metalloproteinase 9 (MMP9)	ACGCAGACATCGTCATCCAG	AACCGAGTTGGAACCACGAC
Interleukin 6 Receptor (IL6R)	CTCCTCTGCATTGCCATTGT	TGTGGCTCGAGGTATTGTCA
Osteocalcin (BGLAP)	TCAGCCAACTCGTCACAGTC	GGTGCAGCCTTTGTGTCC
Osteopontin (SPP1)	AACGCCGACCAAGGAAAACT	GCACAGGTGATGCCTAGGAG
Receptor activator of nuclear factor kappa-B (RANK)	TGCCTTGCAGGCTACTTCTC	AACATGGGGTTCATTTGGTGG
runt-related transcription factor 2 (RUNX2)	CGCCTCACAAACAACCACAG	ACTGCTTGCAGCCTTAAATGAC
Toll-like receptor 4 (TLR4)	GGTCAGACGGTGATAGCGAG	TTTAGGGCCAAGTCTCCACG
β-Actin	CTTCCTGGGCATGGAGTC	AGCACTGTGTTGGCGTACAG

Primer pairs were purchased from Merck KGaA (Darmstadt, Germany)

### Graphical illustration and statistics

**Fig. 1:**
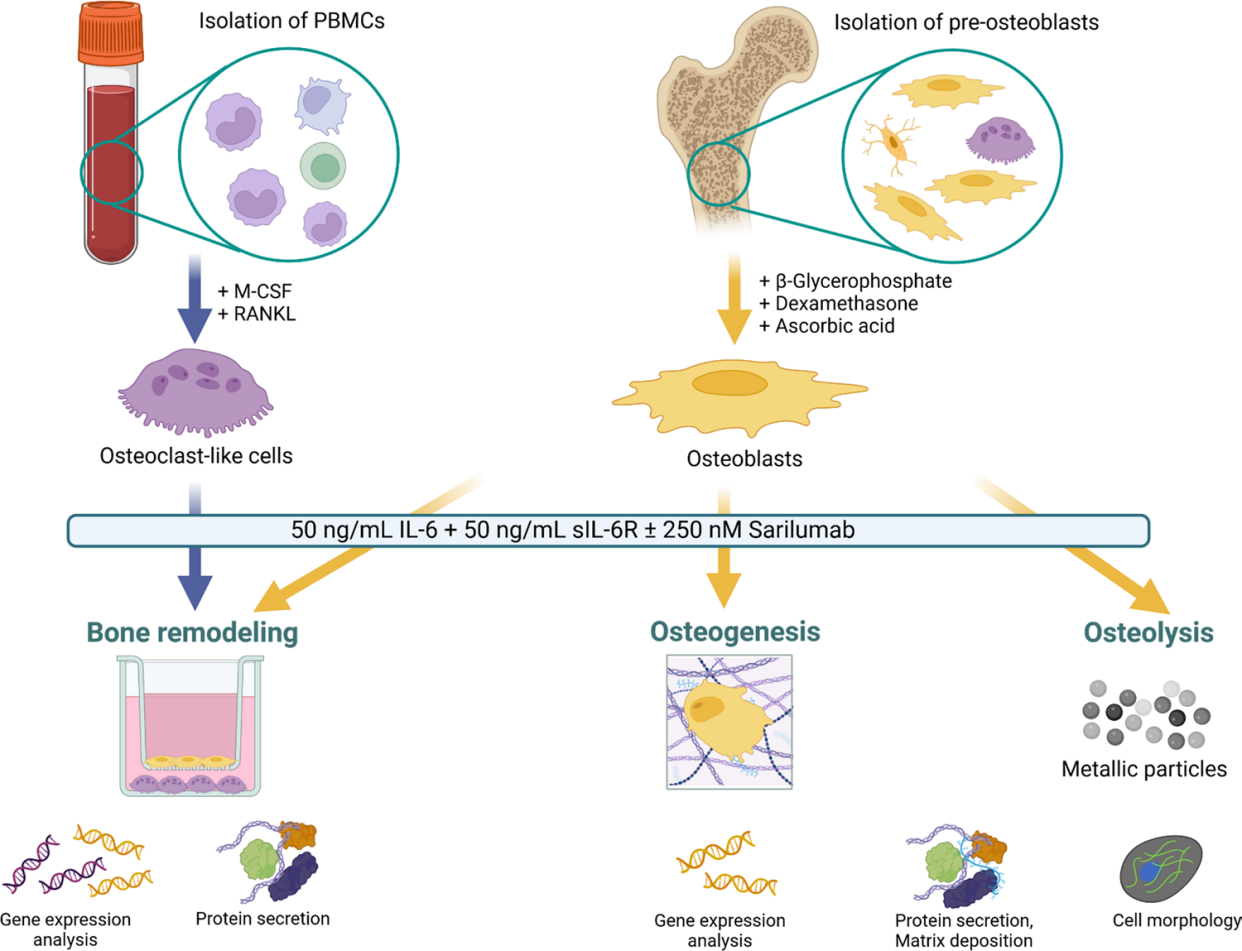
Schematic overview of the experimental setup and the methods used. The figure was created on 11/09/22 at 09:10 AM with biorender (https://biorender.com)

Figure 1 shows the workflow of the experimental studies. All experiments were performed with osteoblasts from seven individual RA donors (male, n = 3: 68.3 years ± 4.2 years; female, n = 4: 72.3 years ± 4.9 years). Co-cultures were performed with osteoblasts and PBMCs from three different donors (1 male, 65 years; 2 females, 69.5 ± 0.5 years). If not otherwise stated, data were presented as individual values with median and interquartile ranges, and statistical analysis was performed using GraphPad Prism, version 8.0 (GraphPad Software, San Diego, CA, USA). The normality of the data was proven via the Shapiro–Wilk test. Different stimulation groups were compared using repeated measures two-way ANOVA with Bonferroni's multiple comparison post hoc test, as required. In the co-culture experiments, data were analyzed separately in co-cultures and monocultures with either repeated measure one-way ANOVA with Bonferroni's multiple comparison post hoc test or with Friedman's test with Dunn's post hoc test. Comparisons between co-cultured and monocultured cells by repeated measures two-way ANOVA were only performed when this was feasible, i.e., the analyzed marker was only secreted or expressed by one of the two tested cell types. A *p*-value of less than 0.05 was defined as statistically significant.

## Results

### Secretion of IL-6, sIL-6R, and sgp130 by human osteoblasts derived from RA patients

**Fig. 2:**
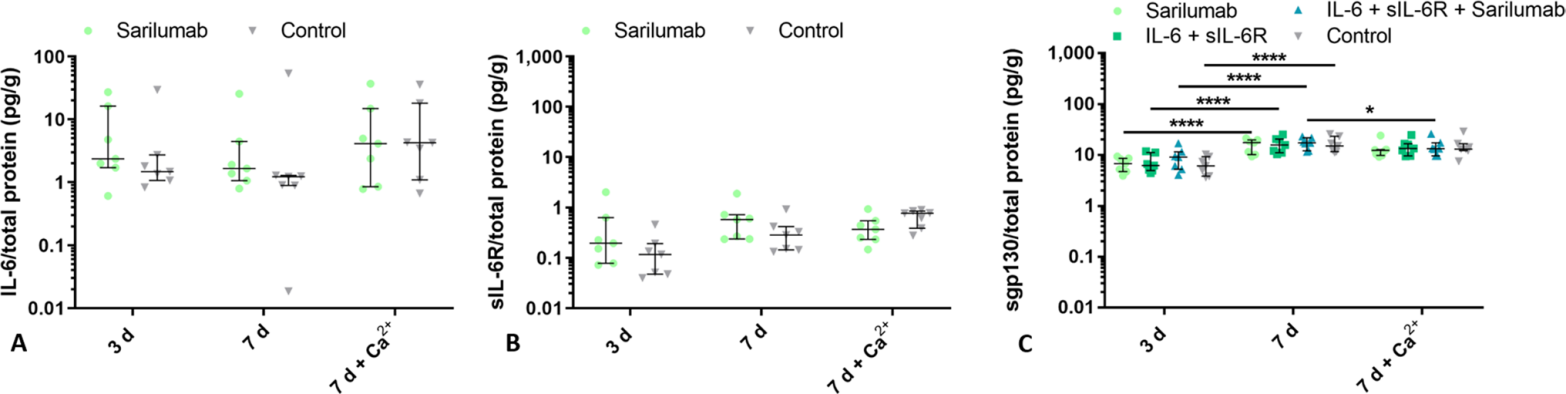
Basal protein release of IL-6, sIL-6R, and sgp130*.* The baseline levels of IL-6 (**A**), sIL-6R (**B**), and sgp130 (**C**) of human osteoblasts over 3 and 7 days in calcium-free or calcium-supplemented medium following treatment with and without sarilumab were determined. Data are presented as individual values with median and interquartile ranges (n = 7). Statistical significance was determined with a 2-way ANOVA and Bonferroni multiple comparison post hoc test: *p < 0.05; ****p < 0.0001

First, baseline levels of secreted IL-6 and sIL-6R were determined from osteoblasts treated with sarilumab for three and seven days (Fig. 2A, B). The results indicate a high donor-specific variation in the secretion of IL-6; however, the addition of sarilumab did not influence the individual secretion. Also, no changes were demonstrated by the addition of calcium to the cell culture medium or the cultivation time. The sIL-6R release was lower compared to IL-6 secretion. There were also no time-dependent changes in the secretion of sIL-6R, nor did the addition of calcium influence protein concentration in the supernatant. More interestingly, osteoblasts secreted sgp130 to a higher extent (Fig. 2C). In calcium-free medium, the release of sgp130 was significantly increased in all stimulation groups after seven days compared to three days (all: *p* < 0.0001). No significant differences were found between the different stimulations. Cells stimulated for seven days with IL-6 + sIL-6R + sarilumab in calcium-supplemented medium showed significantly decreased sgp130 concentration compared to the calcium-free stimulation group (*p* = 0.0339).

### Osteoblastic viability and morphology and gene expression

**Fig. 3:**
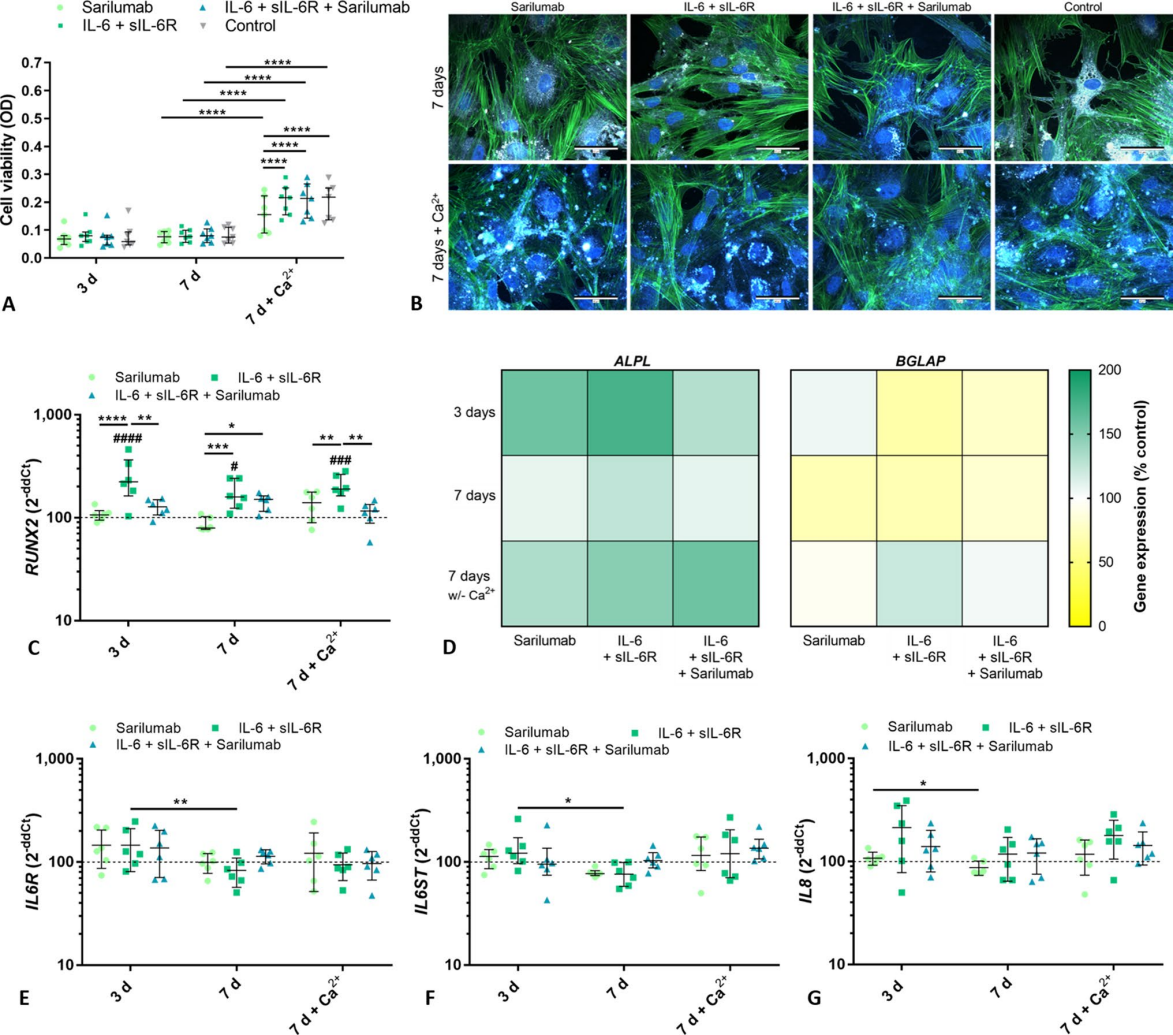
Influence of sarilumab, IL-6 + sIL-6R, and IL-6 + sIL-6R + sarilumab on osteoblastic viability, morphology, and differentiation capacity. Cell viability (**A**), cell morphology (**B**), and gene expression of osteogenic differentiation markers (**C**–**G**) of human osteoblasts isolated from RA patients stimulated with (i) sarilumab, (ii) IL-6 + sIL-6R, (iii) IL-6 + sIL-6R + sarilumab and referred to unstimulated control. **A** WST-1 assay was performed to determine cell viability. **C**–**G** Total RNA of osteoblasts was isolated, and relevant genes of osteogenic differentiation were examined by qPCR. The results were calculated by the 2^−ΔΔCT^ method and normalized to the unstimulated control. The results were shown as median within the heatmaps or individual values with median and interquartile ranges within diagrams (n = 7). Statistical significance was determined using the 2-way ANOVA and Bonferroni multiple comparison post hoc test: *p < 0.05; **p < 0.01; ***p < 0.001; ****p < 0.0001 (significance between stimulation groups); ^#^*p* < 0.05; ^##^*p* < 0.01; ^###^*p* < 0.001; ^####^*p* < 0.0001 (significance to control)

Effects on osteoblastic viability, cell morphology, and gene expression were analyzed after the different stimulations. Whereas in calcium-free medium, cell viability was not affected by the different stimulants nor by the incubation time (Fig. 3A), the addition of calcium led to a significant increase in cell viability (*p* < 0.0001). While the increase was independent of the stimulation, there were also differences between the stimulation groups in the calcium-supplemented medium. Specifically, human osteoblasts treated with sarilumab alone in calcium-containing medium showed significantly lower cell viability compared to the other stimulation groups and the unstimulated cells (*p* < 0.0001).

Incubation of osteoblasts in medium containing calcium resulted in a change in cell morphology (Fig. 3B). The cells showed an altered orientation of their cytoskeleton with circular-arranged actin filaments compared to cells without supplemented calcium, which had elongated actin filaments. Darkfield imaging also revealed bright accumulations in cells incubated with calcium. It is suggested that these bright-shining agglomerates were hydroxyapatite accumulations induced by adding calcium. In sarilumab-treated cells and controls, larger amounts of these accumulations were detected compared to the other stimulation groups.

Stimulation with IL-6 + sIL-6R tended to increase the expression of relevant mRNA transcripts for IL-6 signaling (Fig. 3E, F) and osteogenic differentiation (Fig. 3C, D), particularly on day 3, while on day 7, gene expression mainly returned to control level. However, these effects were not significant apart from the regulation of the osteoblastic transcription factor *RUNX2.* The significant increase in gene expression of *RUNX2* was observed at both time points and was not affected by supplementation with calcium (IL-6 + sIL-6R vs. control 3d: *p* < 0.0001; 7d: *p* = 0.0015; 7d + Ca^2+^: *p* = 0.0005). Addition of sarilumab downregulated the stimulation of *RUNX2* by IL-6 + sIL-6R (IL-6 + sIL-6R + sarilumab vs. IL-6 + sIL-6R 3d: *p* = 0.0018; 7d + Ca^2+^: *p* = 0.0018). The effects of sarilumab treatment alone were comparable to gene expression rates in the untreated controls (Fig. 3C).

### Differentiation capacity of human osteoblasts derived from RA patients

**Fig. 4:**
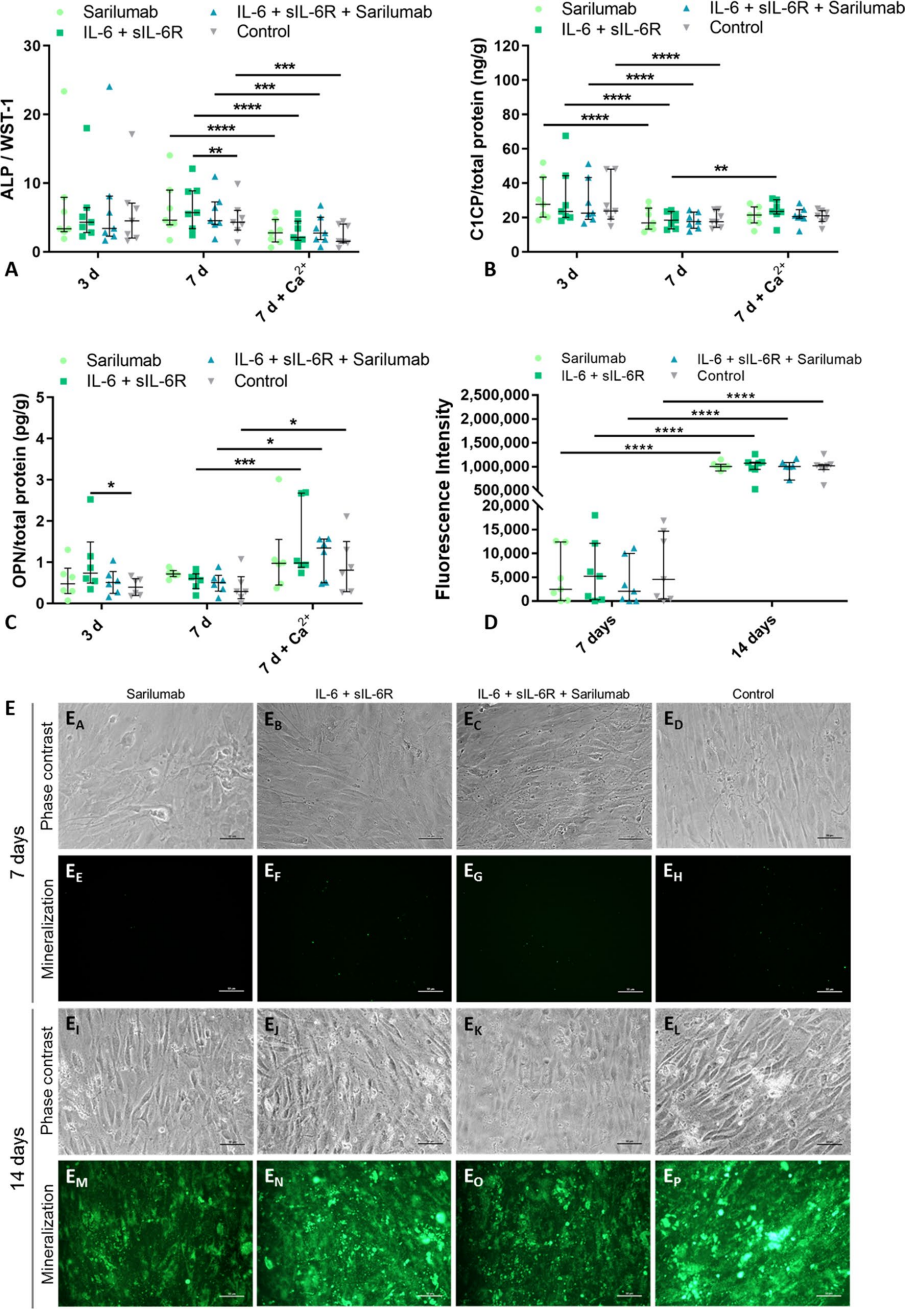
Influence of different stimulation regimes on osteoblastic differentiation and mineralization. **A** Alkaline phosphatase (ALP) activity was quantified by hydrolysis of p-nitrophenyl phosphate. The activity was related to the viability of cells. The release of **B** C1CP and **C** OPN was examined in the supernatants of exposed osteoblasts by ELISA and related to the total protein amount. To investigate the mineralization capacity of cells, the mineralization assay OsteoImage™, which stains hydroxyapatite in deposits, was performed after the cells were treated in calcium-containing medium for 7 or 14 days. Fluorescence intensity was measured at excitation/emission wavelengths of 492/520 nm using a plate reader (**D**). Data are shown as individual values with median and interquartile ranges (n = 7). Statistical significance was determined by a 2-way ANOVA and Bonferroni multiple comparison post hoc test: *p < 0.05; **p < 0.01; ***p < 0.001; ****p < 0.0001. In addition, phase contrast (E: a-d; i-l) and fluorescence images (E: e-m; h-p) were taken. Bar: 50 µm

The enzymatic activity of alkaline phosphatase (ALP, normalized to metabolic activity), as well as the secretion of collagen 1 pro-peptides (C1CP) and osteopontin (OPN), were used to analyze the differentiation capacity of osteoblasts. Furthermore, the deposition of hydroxyapatite was determined (Fig. 4). In general, the addition of calcium to the cell culture medium resulted in a significant decrease in the normalized ALP activity (all: *p* < 0.0001; Fig. 4A) due to the increased metabolic activity of the cells in the calcium-containing medium as shown in Fig. 4A. Furthermore, calcium supplementation elevated the protein secretion of C1CP and OPN when comparing data in calcium-free and calcium-supplemented cell culture medium at day 7 of cultivation (Fig. 4; 7 d vs. 7 d + Ca^2+^; C1CP: IL-6 + sIL-6R: *p* = 0.0066; OPN: IL-6 + sIL-6R: *p* = 0.0003; IL-6 + sIL-6R + sarilumab: *p* = 0.0149; control: *p* = 0.0431). There was also an effect of incubation time on C1CP secretion with significantly lower protein release at day 7 compared to day 3 in calcium-free medium (all groups: *p* < 0.0001).

When analyzing the influence of stimulants and inhibitors of IL-6 signaling on these differentiation markers, significant changes were observed in calcium-free medium for stimulation with IL-6 + sIL-6R compared to untreated cells for ALP activity at day 7 (*p* = 0.0062) and OPN secretion at day 3 (*p* = 0.0435). There was also no influence of the stimulants and inhibitors on mineralization capacity. While there was a significant accumulation of hydroxyapatite over time (7 days vs. 14 days: *p* < 0.0001 for all stimulation groups), which was determined by the quantification of the fluorescence intensities (Fig. 4D) and confirmed by light and fluorescence microscopy (Fig. 4E), there were no differences due to the stimulation groups.

### Influence of IL-6/sIL-6R and sarilumab in co-cultures of osteoblasts and osteoclast-like cells

Since bone remodeling processes involve several cell types, the influence of stimulants and inhibitors of IL-6 signaling was investigated in co-cultures of osteoblasts and osteoclasts from the same patient for three independent donors. The co-cultures were stimulated for seven days, and endpoints with regard to cell vitality and differentiation capacity were assessed in the respective groups.

#### Osteoblasts

**Fig. 5:**
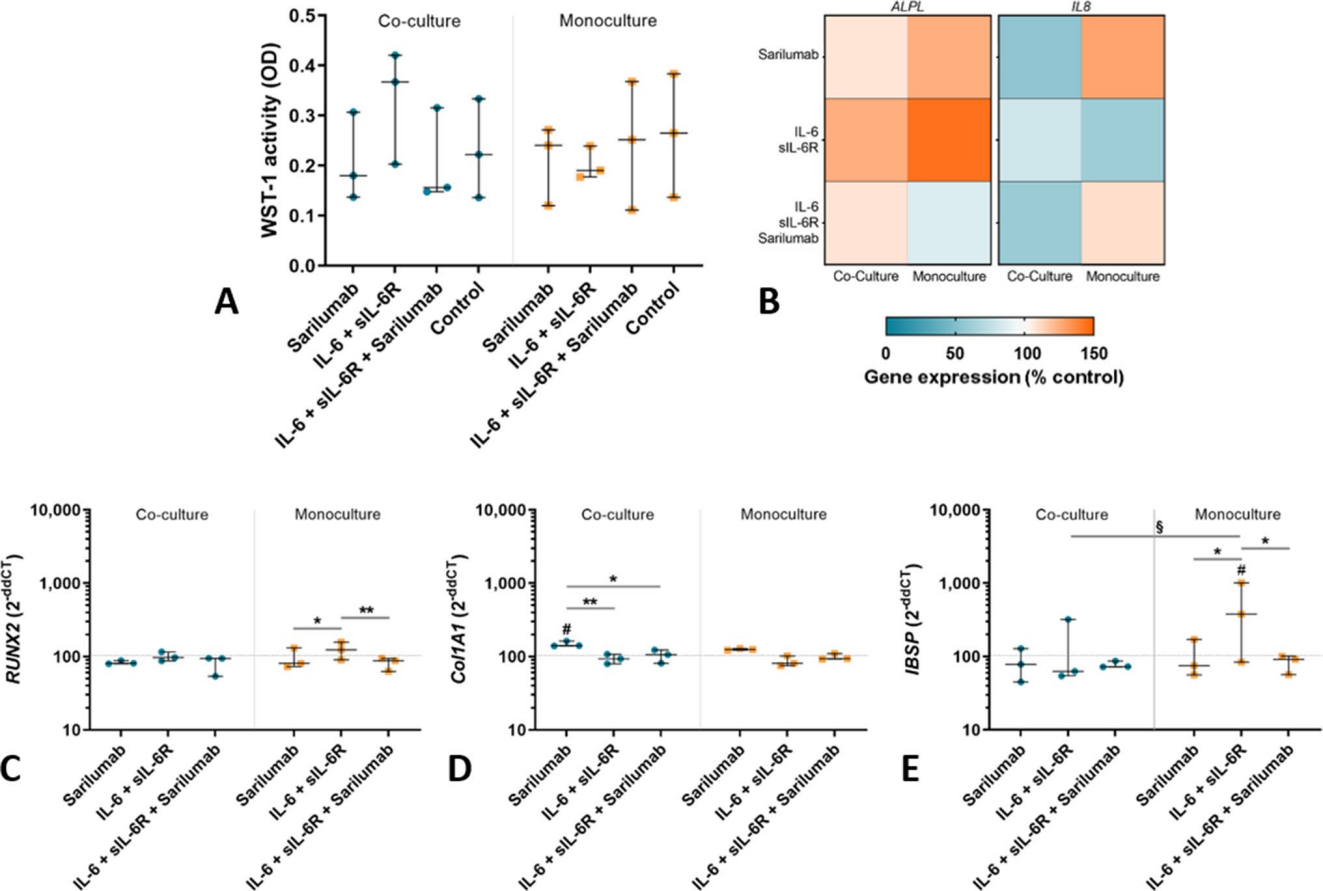
Influence of sarilumab, IL-6 + sIL-6R, and IL-6 + sIL-6R + sarilumab treatment in osteoblasts co-cultured with osteoclast-like cells. (**A**) The viability of osteoblasts in co-culture and osteoblasts monoculture was determined via WST-1 assay after cells were treated with (i) sarilumab, (ii) IL-6 + sIL-6R, (iii) IL-6 + sIL-6R + sarilumab and iv) unstimulated after seven days. (**B**–**E**) Gene expression of ALPL and IL8 (B), RUNX2 (**C**), Col1A1 (**D**), and IBSP (**E**) was determined via qRT-PCR. Results were analyzed using the 2^−ΔΔCt^ method (% unstimulated control). The results were shown as median within the heatmaps or individual values with median and interquartile ranges within diagrams (n = 3). Statistical significance was determined using a repeated measures 2-way ANOVA and Bonferroni multiple comparison post hoc test: *p < 0.05; **p < 0.01 (significance between stimulation groups); ^#^*p* < 0.05 (significance to control); ^§^*p* < 0.05 (significance between co-culture and monoculture)

The viability of co-cultured osteoblasts showed a statistical trend with regard to the influence of treatment on viability (*p* = 0.0898); however, in the post hoc tests, no significant differences between the different stimulation groups were observed (Fig. 5A).

Gene expression after stimulation with IL-6 signaling molecules was mainly unaltered in the co-cultured cells. Whereas the significant upregulation in *RUNX2* gene expression was confirmed in the monocultured osteoblasts following stimulation with IL-6 + sIL-6R compared to either the sole sarilumab (*p* = 0.0421) or IL-6 + sIL-6R + sarilumab (*p* = 0.0055) treatment, *RUNX2* gene expression was unchanged when co-cultured with osteoclasts (Fig. 5C). Also, *IBSP* mRNA was upregulated in the monoculture following stimulation with IL-6 + sIL-6R (IL-6 + sIL-6R vs. control: *p* = 0.0281; IL-6 + sIL-6R vs. sarilumab: *p* = 0.0183; IL-6 + sIL-6R vs. IL-6 + sIL-6R + sarilumab: *p* = 0.0183); however, this effect was attenuated in the co-culture (IL-6 + sIL-6R co-culture vs. monoculture: *p* = 0.0213; Fig. 5E). Interestingly, as the only exception *Col1A1* gene expression was influenced in the co-cultures. *Col1A1* mRNA was significantly induced in co-cultured osteoblasts following administration of sarilumab compared to unstimulated cells (*p* = 0.0134) as well as the stimulation with IL-6 + sIL-6R (*p* = 0.0053) or IL-6 + sIL-6R + sarilumab (*p* = 0.0169). In monocultures, *Col1A1* gene expression was also upregulated by sarilumab compared to IL-6 + sIL-6R, however, this effect did not reach significance (*p* = 0.0555).

#### Osteoclast-like cells (OLCs)

**Fig. 6:**
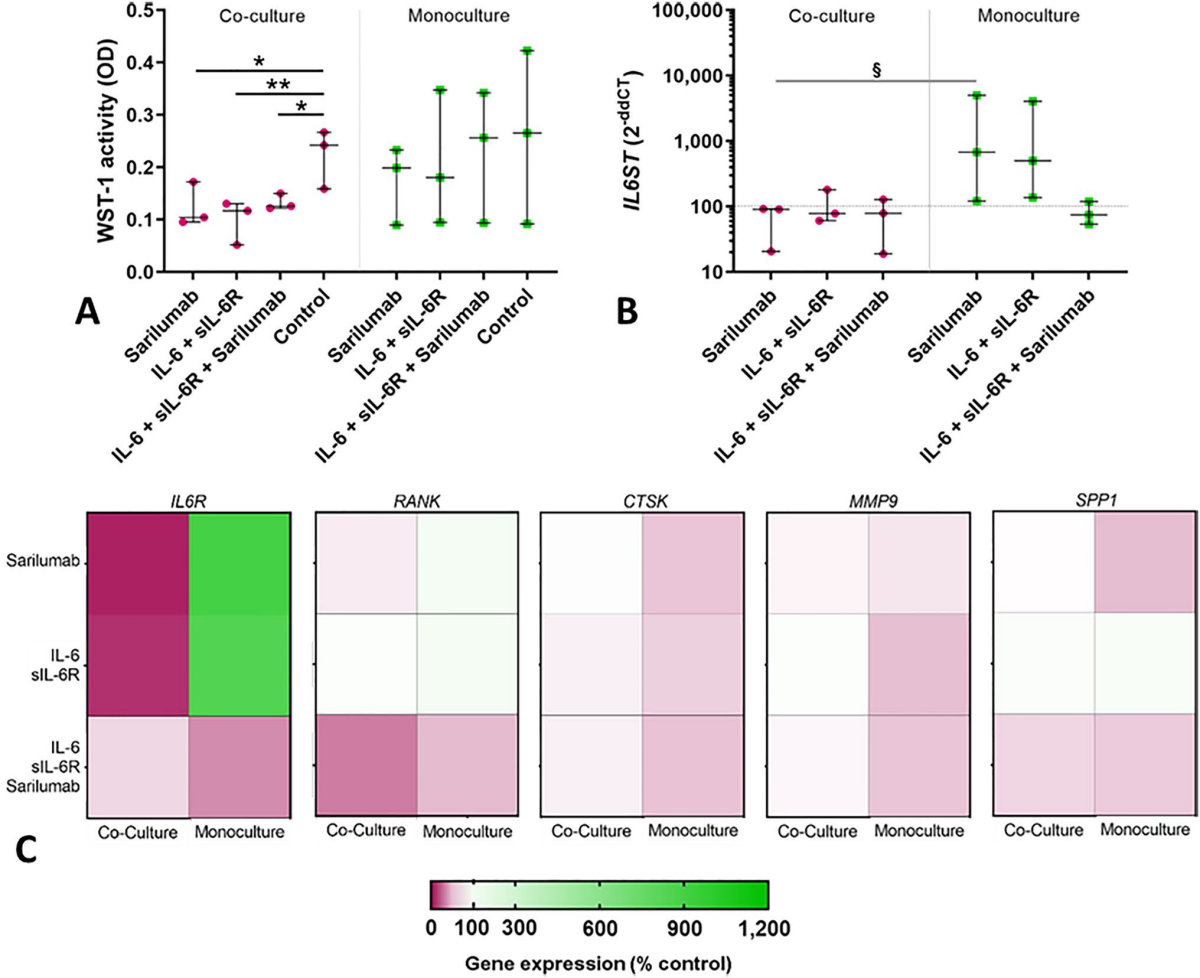
Influence of sarilumab, IL-6 + sIL-6R, and IL-6 + sIL-6R + sarilumab treatment in human osteoclast-like cells co-cultured with osteoblasts. OLCs co-cultured with osteoblasts and OLCs in monoculture were treated with (i) sarilumab, (ii) IL-6 + sIL-6R, (iii) IL-6 + sIL-6R + sarilumab. After seven days, cell viability was determined via WST-1 (**A**), and the stimulation effects on gene expression of IL6ST (**B**), IL6R, RANK, CTSK, MMP9, and SPP1 (**C**) were assessed by qPCR. Results were quantified by the 2^−ΔΔCt^ method and related to the untreated control. The results were shown as median within the heatmaps or individual values with median and interquartile ranges within diagrams (n = 3). Statistical significance was determined using repeated measure one-way ANOVA with Bonferroni's multiple comparison post hoc test when comparing groups within either the co-culture or monoculture setup: *p < 0.05; **p < 0.01 (significance to control) and a 2-way ANOVA and Bonferroni multiple comparison post hoc test when comparing co-culture and monoculture: ^§^*p* < 0.05 (significance between co-culture and monoculture)

The viability of co-cultured OLCs was significantly reduced after the different treatments compared to untreated cells (sarilumab: *p* = 0.0256, IL-6 + sIL-6R: *p* = 0.0086, IL-6 + sIL-6R + sarilumab: *p* = 0.0395, all vs. control; Fig. 6A).

OLCs cultured with osteoblasts showed slightly decreased expression of *IL6ST* in all stimulation groups compared to untreated cells. While treatment of monocultured OLCs with sarilumab or IL-6 + sIL-6R resulted in increased expression of *IL6ST*, treatment with IL-6 + sIL-6R + sarilumab resulted in decreased mRNA levels compared to untreated cells. After stimulation of cells with sarilumab, a significant increase (*p* = 0.0489) in *IL6ST* mRNA levels was detected in monocultures compared to co-cultured OLCs (Fig. 6B).

While gene expression of *IL6R*, *RANK*, *CTSK*, *MMP9,* and *SPP1* was also analyzed in OLCs, no significant changes were observed, neither due to stimulation or inhibition of IL-6 signaling nor due to culture conditions such as co- or monoculture.

#### Protein secretion in co-cultures

The protein levels of C1CP, OPN, IL-8, and sgp130 in the supernatants of the co-cultured and monocultured cells were determined (Table 3). Contrary to the gene expression data, the release of C1CP by osteoblasts was not affected by the different treatments in the co-culture experiment. Moreover, no differences were detected between co-culture and monoculture. Comparing the IL-8 concentrations in the co-culture with the monocultures, it is noticeable that osteoblasts exhibited a high synthesis rate and the majority of IL-8 protein in the co-culture supernatants is likely to be derived from osteoblasts, whereas OLCs showed only a low synthesis rate. No significant differences between the stimulations were detected. OPN, on the contrary, was mainly secreted by OLCs. While it appears that the co-culture had a synergistic effect on OPN concentrations in the supernatants compared to the additive secretion from osteoblasts and OLCs in monoculture, this effect was not significant. Monocultured OLCs secreted lower levels of sgp130 than monocultured osteoblasts. In the latter cells, treatment with IL-6 + sIL-6R resulted in a significantly increased sgp130 secretion compared to unstimulated cells (*p* = 0.0420). There were no differences in the sgp130 concentration in the supernatants from the co-culture, and the observed synergistic effect was again not significant.

**Table 3:** Secretion of different proteins of co-cultured human osteoblasts (hOB) and human osteoclast-like cells (OCL) compared to monocultures following stimulation with sarilumab, IL-6 + sIL-6R and IL-6 + sIL-6R + sarilumab

Protein	Co-Culture					Monoculture			
	Sarilumab	IL-6/sIL-6R	IL-6/sIL-6R Sarilumab	Control		Sarilumab	IL-6/sIL-6R	IL-6/sIL-6R Sarilumab	Control
C1CP [ng/mg]	10.3 ± 1.8	12.0 ± 2.2	12.5 ± 2.7	11.3 ± 2.6		12.6 ± 3.2	12.8 ± 2.7	11.8 ± 3.0	11.9 ± 3.2
IL-8 [pg/mg]	105 ± 70	131 ± 97	135 ± 94	138 ± 97	hOB	152 ± 119	157 ± 127	158 ± 126	129 ± 101
					OLCs	13 ± 3	16 ± 4	13 ± 1	16 ± 4
OPN [pg/mg]	662 ± 41	652 ± 110	756 ± 72	723 ± 70	hOB	103 ± 50	88 ± 44	91 ± 51	82 ± 46
					OLCs	325 ± 35	407 ± 99	369 ± 80	368 ± 60
sgp130	15.7 ± 2.3	17.8 ± 3.6	20.7 ± 4.1	16.5 ± 3.3	hOB	9.2 ± 1.7	11.6 ± 0.7*	8.8 ± 1.4	6.6 ± 1.8
					OLCs	1.8 ± 0.3	1.5 ± 0.3	1.5 ± 0.3	1.5 ± 0.2

Collagen type 1 pro-peptide (C1CP), interleukin (IL-)8, osteopontin (OPN), and soluble gp130 (sgp130) were determined in the supernatants via ELISA and related to total protein. Data are presented as mean values ± SEM (n = 3). Statistical significance was determined using repeated measure one-way ANOVA with Bonferroni's multiple comparison post hoc test when comparing groups within either the co-culture or monoculture setup: *p < 0.05 (significance to control)

#### Effects of particle exposure

The influence of CoCr28Mo6 particle exposure on osteoblasts of RA patients stimulated with IL-6 + sIL-6R ± sarilumab was investigated. In addition, there was also a sole treatment with and without sarilumab. Since the dissolved particles were stored in ethanol, an appropriate solvent control was included.

**Fig. 7:**
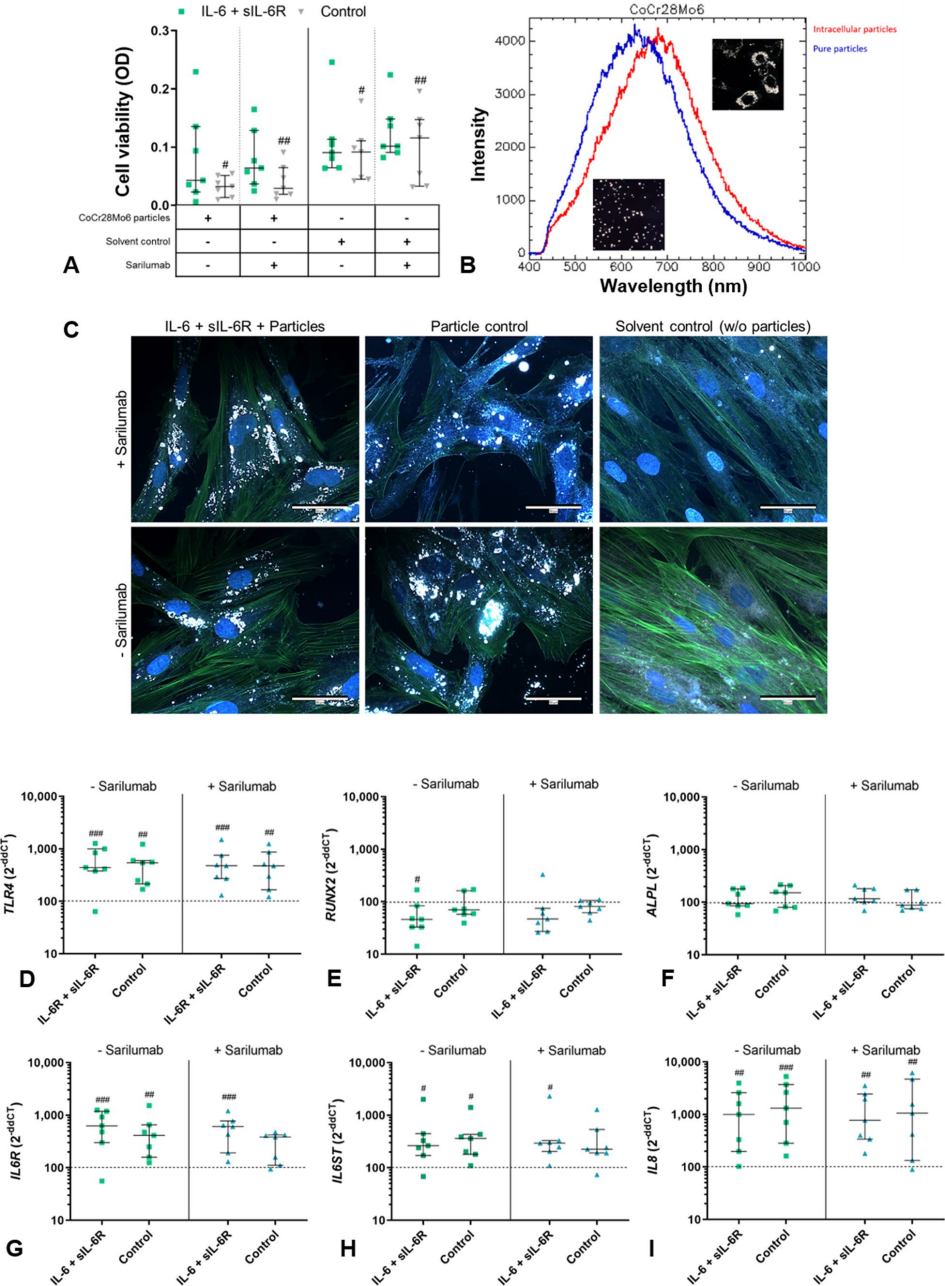
Influence of CoCr28Mo6 particle exposure to human osteoblasts stimulated with IL-6 + IL-6R ± sarilumab. Before osteoblasts were treated with particles for 48 h, they were first stimulated for 24 h with (**i**) sarilumab, (ii) IL-6 + sIL-6R, and (iii) IL-6 + sIL-6R + sarilumab. After a total incubation period of 72 h, cellular activity was determined using a WST-1 (**A**). Hyperspectral images of CoCr28Mo6 particles in solution (blue) and after incubation with cells (red) were taken with the CytoViva® microscope system, and the mean spectral profiles are shown (**B**). In addition, combined fluorescence darkfield images (**C**) of the different stimulations were taken. The actin cytoskeleton of the cells was stained with phalloidin, and the nuclei were counterstained with DAPI (**C**). Furthermore, gene expression of *TLR4*, *RUNX2*, *ALPL*, *IL6R*, *IL6ST*, and *IL8* was examined. Results were quantified using the 2^−ΔΔCt^ method (**D**–**I**). Within diagrams, results are depicted as individual values with median and interquartile ranges (n = 7). Statistical significance was determined using a 2-way ANOVA and Bonferroni multiple comparison post hoc test: ^#^*p* < 0.05; ^##^*p* < 0.01; ^###^*p* < 0.001; ^####^*p* < 0.0001 (significance to control)

Exposure to metallic particles generally resulted in reduced cell viability, and in control as well as sole treatment with sarilumab, the reduction was significant (control: *p* = 0.0193; sarilumab: *p* = 0.0083; Fig. 7A). Stimulation with IL-6 + sIL-6R ± sarilumab partly attenuated the loss of cell viability (Fig. 7A). As there were no differences between stimulation groups in the absence of particles, our results suggest that IL-6 + sIL-6R stimulation specifically counteracted the detrimental effects of particle exposure.

In addition, we looked at whether osteoblasts ingested the particles and whether this affected the actin cytoskeleton. For the analysis, hyperspectral imaging was performed using the CytoViva® hyperspectral darkfield microscope system. First, the hyperspectral data of the particle solution were recorded in the wavelength range from 400 to 1000 nm. A specific peak of 625 nm was found for the CoCr28Mo6 particles, and specific mapping was used to identify the corresponding particles in the cells. This resulted in a red shift of the particles towards about 680 nm. The change to the higher wavelengths indicated an absorption into the cells (Fig. 7B).

Imaging of the actin cytoskeleton using the fluorescence module of the hyperspectral dark-field microscope confirmed the results of the hyperspectral analyses (Fig. 7C). The staining of the actin cytoskeleton of the osteoblasts visualized that the actin filaments stretched over the particles. Moreover, it seems that the cytoskeleton was not affected by the particles. Regarding the different stimulations, particle uptake appeared to be reduced when osteoblasts were stimulated with IL-6 + sIL-6R, compared to the particle uptake of cells stimulated with IL-6 + sIL-6R + sarilumab.

Particle exposure significantly increased *TLR4* gene expression rates compared to samples incubated without particles (IL-6 + sIL-6R: *p* = 0.0005, control *p* = 0.0013, IL-6 + sIL-6R + sarilumab *p* = 0.0009, sarilumab *p* = 0.0017). However, no differences could be detected between the different stimulations (Fig. 7D). Expression of *RUNX2* was significantly decreased after exposure to particles and IL6 + sIL6R (*p* = 0.0366). The other stimulation groups also showed reduced mRNA levels compared to osteoblasts stimulated without particles (Fig. 7E). *ALPL* mRNA was unaffected by exposure to particles and under the respective stimulations (Fig. 7F).

The exposure to CoCr28Mo6 particles also significantly increased gene expression of *IL6R*, *IL6ST*, and *IL8* compared to the solvent control (Fig. 7G–I). Stimulation with IL-6 + sIL-6R did not mitigate this effect, and no differences were observed between the stimulation groups.

Protein secretion of C1CP, IL-8, and sgp130 was further examined in the supernatants of particle-treated cells and the solvent control. Data of secreted proteins are summarized in Table 4. The concentration of C1CP was significantly decreased after treatment of cells with particles compared to the solvent control (IL-6 + sIL-6R: *p* = 0.0014; control: *p* = 0.0034; IL-6 + sIL-6R + sarilumab: *p* = 0.0002). No differences were detected between the stimulations. A clear increase in IL-8 secretion was determined for particle-exposed cells stimulated with IL-6 + sIL-6R. The additional treatment with sarilumab led to a noticeable reduction in protein secretion. In osteoblasts that were not additionally stimulated with IL-6 + sIL-6R, treatment with sarilumab had no effect on the release of IL-8. In general, exposure of osteoblasts to particles resulted in reduced release of sgp130, and the reduction was significant except for stimulation with IL-6 + sIL-6R (control: *p* = 0.0028; IL-6 + sIL-6R + sarilumab: *p* = 0.0004; control + sarilumab: *p* = 0.005). Moreover, significantly increased sgp130 concentrations were detected without the additional treatment with sarilumab (IL-6 + sIL-6R vs. sarilumab *p* = 0.0023; control vs. sarilumab *p* = 0.0295).

**Table 4:** Secretion of different proteins of particle-exposed human osteoblasts stimulated with IL-6 + sIL-6R ± sarilumab

Protein (%)	− Sarilumab		+ Sarilumab	
	IL-6 + sIL-6R	Control	IL-6 + sIL-6R	Control
C1CP	49 (33; 58)^###^	50 (36; 65)^##^	40 (26; 171)^###^	51 (43; 231)
IL-8	141 (127; 161)	116 (87; 387)	88 (56; 119)	107 (44; 198)
sgp130	91 (0; 107)^a^	79 (0; 112)^#,b^	64 (35; 95)^###^	64 (43; 90)^##,a,b^

The proportion of collagen type 1 pro-peptide (C1CP), interleukin (IL-)8, and soluble gp130 (sgp130) per total protein was determined in the supernatants via ELISA and related to the concentration of secreted proteins from cells of the solvent control. Data are presented as mean values ± SEM (n = 7). Statistical significance was determined using a 2-way ANOVA and Bonferroni multiple comparison post hoc test: ^#^*p* < 0.05; ^##^*p* < 0.01; ^###^*p* < 0.001 (significance to solvent control); ^a^p < 0.01, ^b^p < 0.05 (significance to sarilumab treatment)

## Discussion

### Influence on differentiation and morphology

Since IL-6 is a key cytokine in the pathogenesis of rheumatoid arthritis, its effect on osteoblasts of RA patients was investigated initially. IL-6 can interact with cells only in the presence of soluble IL-6R, so IL-6 was used in combination with sIL-6R for treatment. The influence of different concentrations of the cytokine and its receptor on the cells was investigated in a previous study [11]. In this study, it was shown that only high concentrations of 50 ng/mL each for IL-6 and sIL-6R caused an effect; therefore, these concentrations were used in the present study. The IL-6R blocker sarilumab was approved for the treatment of RA several years ago. Sarilumab is approved for the treatment of adult patients with moderate to severe RA and can also be used when standard therapy with MTX does not work or in cases of intolerance [7, 21, 22]. In this study, the effect of sarilumab alone or in combination with IL-6 + sIL-6R on osteoblasts and osteoclasts of RA patients was examined to analyze the cellular response to this medication.

In human serum, high concentrations of sgp130 and sIL-6R were detected, which may have a buffering function on high circulating IL-6 concentrations [23, 24]. In this regard, on the one hand, the trans-signaling of IL-6 can be promoted by the complex formation of IL-6 + sIL-6R. On the other hand, the binding of sgp130 to the IL-6 + sIL-6R complex can inhibit the trans-signaling [23, 24]. The data of our study showed that osteoblasts from RA patients synthesized IL-6, sgp130, and, in small amounts, sIL-6R. No effect of sarilumab on the intrinsic release was observed. Since a higher concentration of sgp130 was detected here, it can be assumed that classical IL-6 signaling predominates in our studied cells. However, it was also reported that the buffering function of sgp130 and sIL-6R has only a minor impact, as fewer IL-6 + sIL-6R ± sgp130 -complexes are formed than previously thought. Thus, classical signaling would predominate even under sIL-6R excess [25].

Regarding the influence of IL-6 on osteoblast differentiation, there are contradictory data from previous studies. On the one hand, it was shown that IL-6 + sIL-6R induced osteoblastic differentiation [4, 11, 26], while other studies reported that the differentiation of cells was impaired under the influence of IL-6 [27–29]. Kaneshiro et al. [28] demonstrated that IL-6 could affect osteoblast differentiation via gp130 through three different signaling pathways. They explained the contradictory data of the studies by indicating that a particular intracellular signaling pathway predominates depending on the stage of cell differentiation and culture conditions. In this study, examination of osteoblast differentiation markers revealed that IL-6 induced signal transduction of osteoblast differentiation via increased *RUNX2* expression. The addition of sarilumab appeared to reduce the induction of differentiation triggered by IL-6 + sIL-6R. This was in clear contrast to our previous study, where we used osteoblasts isolated from femoral heads of patients with primary osteoarthritis. In these cells, stimulation with IL-6 and sIL-6R did not affect RUNX2 expression, which was detected via transcriptome analyses. However, a significant impact on osteogenic differentiation capacity was apparent for these cells, with the induction returning to the level of unstimulated cells after the addition of sarilumab [11]. An explanation for the effect of IL-6 + sIL-6R on osteoblast differentiation observed in this study is given by the study of Malysheva et al. [30]. They demonstrated that the inhibition of osteoblast differentiation by IL-6 is mediated by TNF-α-induced Dickkopf-related protein 1 (DKK-1) and that knockout of IL-6 protected osteogenesis. They hypothesized that IL-6 is a mediator of inhibition of osteoblastic differentiation by TNF-α, which is mainly secreted by T-cells and activated macrophages in rheumatic tissue [2, 30].

In patient studies, monotherapy with sarilumab, i.e., the blocking of IL-6 signaling, resulted in an increased concentration of osteocalcin in serum [31]. Concurrently, IL-6 can promote the bioactivation of osteocalcin [32], thus suggesting contrasting effects of IL-6 signaling on osteocalcin. However, neither stimulation with IL-6 + sIL-6R nor inhibition with sarilumab significantly influenced *BGLAP* expression at the cellular level in our in vitro study. This is consistent with the data from the mineralization experiments since no adverse effect on mineralization was detected.

The addition of sarilumab reduced the expression again. Despite the decreased expression of this marker, no adverse effect on mineralization was detected.

Li et al. [33] demonstrated that osteoblasts increasingly expressed IL-6R during in vitro differentiation; moreover, osteoblasts responded to IL-6 by expressing a non-functional IL-6R [8]. This receptor does not induce signal transduction upon IL-6 binding, so osteoblasts can only respond to IL-6 via sIL-6R and thus only with trans-signaling. Interaction of the IL-6-sIL-6R complex with the gp130 subunit activates the trans-signaling pathway, triggering pro-inflammatory effects. Treatment of cells with IL-6 + sIL-6R resulted in an inflammatory response with increased IL-8 concentrations. It was observed that *IL8* expression was decreased in response to sarilumab, suggesting that sarilumab may inhibit the inflammatory response. Furthermore, Mitsuyama et al. (2004) [34] investigated the effect of calcium on the expression of *IL8* and showed that calcium signaling via NF-κB could regulate the expression of *IL8*. This is consistent with our results that calcium supplementation increased mRNA levels of *IL8*. The reduced IL-8 levels after seven days in calcium-free medium could be due to the increased sgp130 secretion blocking the trans-signaling pathway and thereby causing reduced inflammation.

RA manifests with a loss of bone substance and impaired bone formation, as the inflammatory processes inhibit the activity of osteoblasts while osteoclastic bone resorption is enhanced. However, we were unable to reproduce these effects in our in vitro experiments since the mineralization capacity of osteoblasts was not negatively affected by IL-6, and in combination with calcium in the cell culture, medium matrix formation was even slightly induced after stimulation with IL-6 + IL-6R. It has been previously shown that the reduction of ALP synthesis is induced by hypoxia and acidosis in inflamed synovial tissue. TNF-α reduces bone formation via induction of DKK-1, which inhibits wingless-related integration site (Wnt) signaling [35]. Despite the slight increase in OPN, which can bind calcium ions in its phosphorylated state and thereby inhibits mineralization, mineralization was not affected by stimulation with IL-6 + IL-6R. This could be due to the phosphorylation state of OPN, as OPN dephosphorylated by ALP no longer inhibits mineralization. However, the determination of secretion of OPN by ELISA does not distinguish between the different forms of OPN.

### Influence on bone remodeling

The imbalance between bone formation and resorption in RA is partly caused by chronic inflammation. To better reflect on in vivo situation regarding the influence of IL-6 and sarilumab on bone remodeling, an indirect co-culture of osteoblasts from RA patients and the donor-specific OLCs was performed. Likely, the differing observations between the initial experiments with osteoblasts and the results of the osteoblast monocultures were due to the different culture conditions. Because of the diverse effects of cell culture additives on the differentiation of the different cell types [18], only ascorbic acid and glutamine were added to the medium in the experimental setup for the co-culture experiments. However, as diffusion occurs between media in plates with cell culture inserts, calcium from the RPMI medium was present in these cell cultures.

Our study showed no clear differences in gene expression between mono- and co-cultured osteoblasts after different treatments.

Osteoclasts are known to play a leading role in arthritic bone resorption, with increased activity, longevity, and numbers observed [36]. In this study, co-cultured stimulated OLCs showed reduced viability after the different treatments. It was reported that bone resorption of RA patients is characterized by a high serum concentration of the bone remodeling and resorption markers *MMP-9* [37] and *CTSK* [38] through the synergistic impact of TNF-α, IL-1, and IL-6 [39], whereas IL-6 alone probably plays only a minor role. Consistent with this, no effect of the different treatments on osteoclast resorptive activity was observed in this study. Monocultured OLCs showed increased *IL6ST* expression after sarilumab and IL-6 + sIL-6 treatment. It has been previously reported that OLCs from RA patients have a high surface expression of gp130 and thus can recruit high levels of IL-6/sIL-6Rα [37]. OLCs can recruit more ligands, such as IL-6, due to the increase of gp130 on their cell surface so that the inflammatory response is induced. The decrease in *IL6ST*, as seen after treatment of OLCs with IL-6 + sIL-6R + sarilumab, indicated the inhibition of cell differentiation and thus decreased bone resorption. It is already known that osteoclasts can express IL-6R on their surface. However, the differentiation of osteoclasts from osteoclasts progenitors is not induced via these receptors [40].

While no effect was detected by the addition of the cytokines in this study, it was reported that IL-6 + sIL-6R decreased the expression of RANK in calvaria bone [39]. However, a trend was detected that the expression of *IL6ST*, *IL6R*, and *RANK* responded similarly to the different treatments. This correlation is thought to be indirectly triggered by the regulation of RANKL. The control of the balance of bone formation and bone resorption via RANK-RANKL is shifted in favor of bone resorption in RA. This shift is caused by the interaction of a variety of factors. The release of pro-inflammatory cytokines from tissues stimulates RANKL expression in mesenchymal stem cells and acts directly on osteoclast precursors, further driving osteoclast differentiation and enhancing osteoclastogenesis. IL-6 acts as a pro-osteoclastogenic cytokine by inducing RANKL and stimulating osteoclast progenitors directly via stimulation of gp130 signaling [41].

Axmann et al. [42] described that IL-6R blockade led to a reduction of structural damage and decreased osteoclast formation not by inhibiting the inflammatory response but by interfering with osteoclastogenesis in the joint. The inflammatory response is thereby driven by TNF-α and not by IL-6. The effect of the IL-6-IL-6R complex on osteoclast differentiation has not been fully clarified. In addition to the indirect effect via RANKL, IL-6 may also act directly, in a RANKL-independent manner, on osteoclast formation. The release of IL-6 by osteoblasts or monocytes acts as a stimulator of monocytes, which can then differentiate into osteoclasts. In 1995, it was shown by Flanagan et al. [43] that IL-6 could not replace the osteoclast-inducing effect of stroma; moreover, their results showed that IL-6 alone or in combination with sIL-6R decreased osteoclast formation and bone resorption. They suggested that IL-6 alone does not cause increased bone resorption and that high IL-6 concentrations in combination with IL-6R induce a protective mechanism of the body to counteract excessive bone resorption [43]. Consistent with this, our data showed that IL-6 + sIL-6R resulted in a reduction in osteoclast differentiation and their resorptive activity that was not influenced by the addition of sarilumab nor by culture conditions. Yoshitake et al. (2017) postulated that the effect of IL-6 + sIL-6R on osteoclastic differentiation depends on the disease stage. In the early stage with low RANKL levels, IL-6 + sIL-6R leads to an increasing differentiation of osteoclasts, whereas in the late stage with increased RANKL levels, IL-6 + sIL-6R promote a protective mechanism [44]. Since the isolated donor cells were derived exclusively from older patients with long-term medication, it can be assumed that these cells reflected the later RA stage. This might be the reason why stimulation with IL-6 + sIL-6R did not affect osteoclastogenesis.

Assuming that IL-6 + sIL-6R inhibits osteoclast differentiation and sarilumab blocks IL-6R, and thus IL-6 signaling increased expression of the markers would have been expected. Since it has been shown that the presence of sgp130 can mitigate the effect of IL-6R blockers on osteoclasts [42] and osteoblasts secrete a large amount of sgp130, it can be assumed that the co-culture results were determined by this mechanism.

High levels of OPN are associated with bone destruction due to its ability to bind calcium ions and thus reduce biomineralization. Furthermore, OPN plays a role in the pathogenesis of RA by its ability to promote osteoclast activity indirectly via T-cells. It also has a positive effect on osteoclast migration and adhesion to the bone surface [45, 46]. While at the gene expression level, only minor differences between co-cultured and monocultured cells could be shown, it is clear that osteoclasts secrete high amounts of OPN at the protein level. Monocultured OLCs responded to IL-6 + sIL-6R with increased OPN secretion, which was reduced by adding sarilumab. Co-cultured cells released high levels of OPN after treatment with IL-6 + sIL-6R + sarilumab, whereas *ALPL* expression by osteoblasts was reduced, suggesting that biomineralization by osteoblasts was inhibited in the presence of OLCs. However, this aspect was not considered in this study and should be investigated further in future studies.

### Inhibition of particle-induced inflammation by sarilumab

Despite therapy with DMARDs in combination with biological agents, which are aimed to reduce inflammation and prevent joint destruction, many RA patients require total joint replacement [47]. After primary arthroplasty, RA patients have an increased risk for revision surgery due to higher aseptic loosening rates, as inflammation-mediated osteolysis may be influenced by the systemic inflammatory processes in RA [48, 49]. In recent decades, it has been observed that treatment and patient response to DMARDs resulted in a reduced rate of surgery [47]. In this study, the administration of sarilumab did not affect the particle-induced inflammatory response. Overall, it was shown that treating cells with metallic particles resulted in reduced cell viability. This reduction could already be observed in osteoblasts from osteoarthritic patients in previous studies of our research group [19]. After treatment with IL-6 + sIL-6R + sarilumab, a slight increase in cell viability was shown. Microscopic examinations with a hyperspectral dark-field microscope showed that aggregations of CoCr28Mo6 particles were located on the cell membrane. It is assumed that the particles were phagocytosed by the osteoblasts since the spectral data showed a shift to a higher wavelength. This shift in the spectral peak is probably caused by the agglomeration of the particles [50, 51].

For the detection of danger signals associated with aseptic loosening, TLR4 is considered one of the most relevant receptors [52] and was already demonstrated on the surface of osteoblasts in response to wear particles in previous studies [53]. Increased expression of *TLR4* indicates recognition of particles by cells. It has also been reported that TLRs are overexpressed in various cells during joint inflammation in RA. In this context, activation of the TLR pathway leads to the induction of RANKL expression in osteoblasts, further promoting osteoclastogenesis and bone erosion [36, 54, 55]. Cell-particle interaction triggers an intracellular signaling cascade via NF-kB, leading to increased secretion of cytokines, such as IL-8. The presence of sarilumab seemed to reduce the secretion of IL-8. Examination of differentiation markers clarified that the particles reduced the differentiation capacity of osteoblasts. IL-6 + sIL-6R ± sarilumab appeared to increase the negative effect further, whereas treatment with sarilumab alone resulted in a slight increase of the differentiation marker *RUNX2*. In response to the particles, osteoblasts expressed *IL6R*, and neither IL-6 nor sarilumab affected the expression. Osteoblasts do not express functional IL-6R, so the increased mRNA levels did not result in an anti-inflammatory response via classical IL-6 pathway. The increased mRNA levels of *IL6ST* were not reflected at the protein level, so the trans-signaling pathway could not be inhibited via sgp130, and an inflammatory response was induced via the expression of *IL8*. It is suggested that the protein biosynthesis of osteoblasts was impaired in a particle-induced manner, as they may have a cytotoxic effect on cell organelles [19, 56].

### Influence of the drug pretreatment of RA-patients

RA is a chronic and systemic autoimmune disease that can be treated with the help of various medications. These include glucocorticoids, non-steroidal anti-inflammatory drugs (NSAIDs), and DMARDs. MTX, used as the gold standard for the treatment of RA, is considered a potent stimulator of osteoclast activity and formation and an inhibotor of osteoblast activity [27, 57, 58]. The cells used in these experiments were derived from RA patients who had previously received various drug combinations in combination with prednisolone to treat the disease (Table 1). The prior medication of the patients may be a cause of the results that differed in part from those known from the literature and the donor-specific variations in this study. It is known that glucocorticoid treatment can reduce bone mineral density and promote bone loss by inducing bone resorption and suppressing bone formation [59, 60]. Treatment of pre-osteoblasts with prednisolone resulted in increased ALP activity and decreased calcium deposition, decreased cell viability, and proliferation. It is already known that prednisolone can induce apoptosis of confluent osteoblasts [61]. While cortisol, a glucocorticoid hormone secreted in response to stress, can promote osteoblast differentiation at physiological concentrations [59], there are conflicting data on whether prednisolone can inhibit or increase the biological activity of osteoblasts and osteoclasts. The effect seems to be concentration-dependent and depends on the developmental stage of the osteoblasts [61–63].

Overall, it cannot be completely excluded that the medical treatment of the patients had an impact on the response of the donor cells to the external stimuli. However, the patients stopped their medication two weeks before surgery. It was reported that four days after discontinuation of the drugs, the markers of bone formation had already returned to physiological levels [64].

To maintain the osteogenic phenotype of primary osteoblasts, cells were treated with dexamethasone. The glucocorticoid can inhibit the NF-kB signaling pathway [65], which plays a crucial role in the pathogenesis of RA. Since, in contrast to previous studies [11], only minor effects of the treatments on the cells were shown in this study, it might be that the osteoblasts of the RA patients responded with enhanced sensitivity to re-treatment with a glucocorticoid so that interference may have occurred compared to the experimental treatment of the cells and the expected responses were inhibited. However, NF-kB-dependent responses, such as IL-8 release, were also detected, so this signaling pathway was not completely suppressed. The response of the cells to dexamethasone needs to be investigated in more detail in further studies.

## Conclusion

In summary, our study demonstrated that stimulation with IL-6 and sIL-6R elicits a different molecular and cellular response in osteoblasts from RA patients than cells from patients with osteoarthritis [11]. Thereby, the additional stimulation with sarilumab has minor effects on the cell morphology or differentiation capacity of the cells. Indirect co-cultivation had less impact on the differentiation of donor-specific osteoblasts and osteoclasts in relation to the respective stimulations. The addition of IL-6 and sIL-6R attenuated the effect of CoCr28Mo6 particles on osteoblast survival, but the particle-induced reduction in osteogenic differentiation was not reversed by the respective stimulants. Only a trend toward a reduced release of IL-8 by adding sarilumab in particle-exposed cells could be demonstrated. It cannot be excluded that long-term anti-inflammatory medication directly influenced the phenotype of donor-specific cells. Therefore, further studies are necessary to understand better the molecular impact of such stimulations to derive further targeted therapies in RA patients preventing aseptic implant loosening.

## Data Availability

The datasets used and/or analyzed during the current study are available from the corresponding author upon reasonable request.

## Abbreviations

ALP: Enzymatic alkaline phosphatase
ALPL: Alkaline phosphatase
BGLAP: Bone gamma-carboxyglutamate protein (osteocalcin)
C1CP: C-Terminal pro-peptide of collagen type 1
cDNA: Complementary deoxyribonucleic acid
Col1A1: Collagen type 1
Ct: Cycle threshold
CTSK: Cathepsin K
DAPI: Diamidino-2-phenylindole dihydrochloride
DKK-1: Dickkopf-related protein 1
DMARDs: Disease-modifying anti-rheumatic drugs
DMEM: Dulbecco's Modified Eagle Medium
ENVI: Environment for visualization
FCS: Fetal calf serum
gp130: Signal transducing beta receptor subunit (glycoprotein 130)
HEPES: 2-(4-(2-Hydroxyethyl)-1-piperazinyl)-ethanesulfonic acid
hOB: Human primary osteoblasts
HPRT: Hypoxanthine guanine phosphoribosyl transferase
IBSP: Integrin binding sialoprotein
Ig: Immunoglobulin
IL: Interleukin
IL-6R: Membrane-bound Interleukin-6 receptor
IL6ST: Glycoprotein 130
JAK: Janus kinase
M-CSF: Human macrophage colony-stimulating factor
MMP: Matrix-metalloproteinase
mRNA: Messenger ribonucleic acid
MTX: Methotrexate
NSAIDs: Non-steroidal anti-inflammatory drugs
OLCs: Osteoclast-like cells
OPC: Mononuclear osteoclast progenitor cells
OPN: Osteopontin
PBMC: Human peripheral blood mononuclear cells
PBS: Phosphate-buffered saline
qPCR: Semi-quantitative real-time polymerase chain reaction
RA: Rheumatoid arthritis
RANK: Receptor activator of nuclear factor κB
RANKL: Receptor activator of nuclear factor κB ligand
RNA: Ribonucleic acid
ROI: Region of interest
RPMI: Roswell Park Memorial Institute
RUNX2: Runt-related transcription factor 2
sIL6R: Soluble Interleukin-6 receptor
SPP1: Secreted phosphoprotein 1 (osteopontin)
sRANK: Soluble receptor activator of nuclear factor κB
STAT: Signal transducer and activator of transcription
TBS: Tris-buffered saline
PMSF: Phenylmethylsulfonyl fluoride
TLR: Toll-like receptor
TNF-α: Tumor necrosis factor -α
Wnt: Wingless-related integration site
WST-1: Water-soluble tetrazolium salt
